# FRCS differential attainment related to region and specialty: retrospective cohort study

**DOI:** 10.1093/bjsopen/zrac113

**Published:** 2022-09-19

**Authors:** Osian P James, Katie Mellor, Chris Brown, Richard J Egan, Wyn G Lewis

**Affiliations:** Health Education and Improvement Wales School of Surgery, Tŷ Dysgu, Cefn Coed, Nantgarw, UK; Health Education and Improvement Wales School of Surgery, Tŷ Dysgu, Cefn Coed, Nantgarw, UK; Health Education and Improvement Wales School of Surgery, Tŷ Dysgu, Cefn Coed, Nantgarw, UK; Department of Surgery, Morriston Hospital, Swansea, UK; Swansea University, Singleton Park, Swansea, UK; Health Education and Improvement Wales School of Surgery, Tŷ Dysgu, Cefn Coed, Nantgarw, UK

*Dear Editor*

Fellowship of the Royal College of Surgeons (FRCS) examination first attempt pass rate may be inferred as a surrogate metric of training quality and differential attainment; with the latter aligned to hidden curricula including bias, trainer variability, hospital unit culture, dignity at work, sleep deprivation, and the inverse care law. This study evaluates FRCS first attempt pass rates related to geographical region ( Statutory Education Bodies (SEBs)) and surgical specialty.

The Joint Committee on Intercollegiate Examinations (JCIE) published FRCS pass rates were scrutinized for higher surgical trainees from February 2012 to February 2022^1^. FRCS pass rates were calculated from the number of candidates successful at their first attempt and the total number of candidates sitting the examination. Specialty competition ratios were calculated as the mean of all available data published by Health Education England between 2015 and 2021^2^.

From a total of 17 804 attempts across all 10 specialities and 20 regions categorized by the JCIE, the median pan-specialty FRCS first-pass rates for Section One (S1) and Section Two (S2) were 65.8 per cent and 75.1 per cent respectively. Significant variance was found related to SEB (range S1, Malta 52.2 per cent *versus* Wales 79.2 per cent; S2, Republic of Ireland 70.7 per cent *versus* Severn 84.2 per cent, both *P* < 0.001), see *Table 1*. Regional performance in S1 and S2 correlated strongly (rho 0.048, *P* = 0.026).

**Table 1:** Pan-specialty Fellowship of the Royal College of Surgeons examination first-pass performance by region (February 2012 to 2022)

Region	Section 1		Section 2		Overall		
	Total attempts	Pass rate (%)	Total attempts	Pass rate (%)	Total attempts	Pass rate (%)	Rank
Severn	319	74.9	285	84.2	604	79.3	1
Wales	289	79.2	285	75.8	574	77.5	2
Northern Ireland	235	77.9	217	75.6	452	76.8	3
Oxford	329	70.8	291	81.4	620	75.8	4
South West Peninsula	219	74.4	215	76.3	434	75.3	5
West Midlands	699	69.5	594	78.3	1293	73.5	6
Armed Forces	124	71.8	109	74.3	233	73.0	7
Mersey	400	69.3	372	74.7	772	71.9	8
Yorkshire	208	70.7	221	71.5	429	71.1	9
Scotland	864	67.5	754	74.9	1618	71.0	10
Wessex	337	65.0	284	78.2	621	71.0	11
North Western	639	64.0	507	77.9	1146	70.2	12
Northern	464	68.3	428	71.0	892	69.6	13
East Midlands	470	64.7	418	72.5	888	68.4	14
East of England	601	62.3	525	72.2	1126	66.9	15
South Yorks & Humberside	556	62.8	464	71.8	1020	66.9	16
London	1771	59.7	1441	75.2	3212	66.6	17
Kent, Surrey & Sussex	486	60.1	376	74.5	862	66.4	18
Republic of Ireland	507	61.7	457	70.7	964	66.0	19
Malta	23	52.2	21	71.4	44	61.4	20

First-pass rates related to surgical specialty can be found in *Fig. S1*, with significant variance noted across S1 and S2 (both *P* < 0.001). The highest median (range) first-pass rate in S1 was observed in Trauma and Orthopaedics (79.5 per cent (regional range 64.3–97.3 per cent)) and in S2 by Vascular Surgery (100.0 per cent (33.3–100.0 per cent)), with the lowest in both sections by Oral & Maxillofacial Surgery (S1, 45.9 per cent (33.3–100.0 per cent) and S2, 0.0 per cent (0.0–100.0 per cent)). No association was found between FRCS performance and competition ratios into specialty training (S1, rho –0.273 *P* = 0.446; S2, rho –0.430 *P* = 0.214; *Table S1*).

The principal findings demonstrate that FRCS first-pass performance related to SEB varied by approximately 50 per cent in S1, and 20 per cent in S2. Performance related to surgical specialty varied two-fold in S1, and wholescale from 0–100 per cent in S2, irrespective of the competition ratios associated with specialty training entry. Using data from 2007 to 2017, Brown *et al.* reported almost half the median (range) S2 FRCS first-pass rate (42.1 per cent (26.7–45.6 per cent))^3^. It is a welcome finding that FRCS pass rates have almost doubled, which may be due to enhanced examination-specific focus among trainees and better understanding of the examination process and technique. Yet despite an updated curriculum in 2016, it seems unlikely that these factors alone have contributed to a two-fold improvement in FRCS performance. Inter-specialty variance also seems to be growing; Brown *et al.* reported no significant difference between ranks (*P* = 0.457), but the present study shows a two-fold difference in S1 and wholescale (0–100 per cent) in S2 rank.

The study has inherent limitations. FRCS performance alone takes no account of covert confounding variables including operative ability, academic reach, hospital rota intensity, and other hidden curriculum factors such as burnout, sleep deprivation, dignity at work, geographical deprivation and inequality, and trainer variation. Many other factors have been reported to influence examination performance, including demographics, earlier education, and organizational skills^4^. Interpreting SEB-related examination performance as a surrogate marker of training quality assurance must therefore be made with caution. League tables are contentious, and the product of a finite-game mindset rather than the infinite mindset better associated with success in other competitive arenas^5^. Yet, tables do supply a framework showing accountability and transparency, driving evaluation, competition, clinical effectiveness, and patient safety.

New Joint Committee on Surgical Training curricula should standardize training through uniform annual quality assurance, based on fundamental educational principles, facilitating consensus regarding the international countermeasures required to take the difference out of attainment—an urgent and prescient need.

## Supplementary Material

zrac113_Supplementary_Data

## Data Availability

All data are publicly available on the JCIE website (https://www.jcie.org.uk). The authors are willing to clarify all analytical methods described. The study was not preregistered.
