# Supplementary material for: FRCS differential attainment related to region and specialty: retrospective cohort study
Source: BJS Open. 2022 Sep 19;6(5):zrac113. doi: 10.1093/bjsopen/zrac113 (PMC9487602; doi:10.1093/bjsopen/zrac113)
Supplement: zrac113_Supplementary_Data [file zrac113_supplementary_data.docx]

**FRCS differential attainment related to region and speciality: a retrospective cohort study**

Osian P James^1^, Katie Mellor^1^, Chris Brown^1^, Richard J Egan^2,3^, Wyn G Lewis^1^

1. Health Education and Improvement Wales’ School of Surgery, Tŷ Dysgu, Cefn Coed, Nantgarw, UK, CF15 7QQ.
2. Department of Surgery, Morriston Hospital, Swansea, UK, SA6 6NL.
3. Swansea University, Singleton Park, Swansea, UK, SA2 8PP.

**Correspondence**

Mr Osian James, Health Education and Improvement Wales’ School of Surgery, Tŷ Dysgu, Cefn Coed, Nantgarw, UK, CF15 7QQ. Email: osianpjames@gmail.com.

**Supplementary Materials – Index**

**Supplementary Figures**

Figure S1 Page 2

**Supplementary Tables**

Table S1 Page 3

*
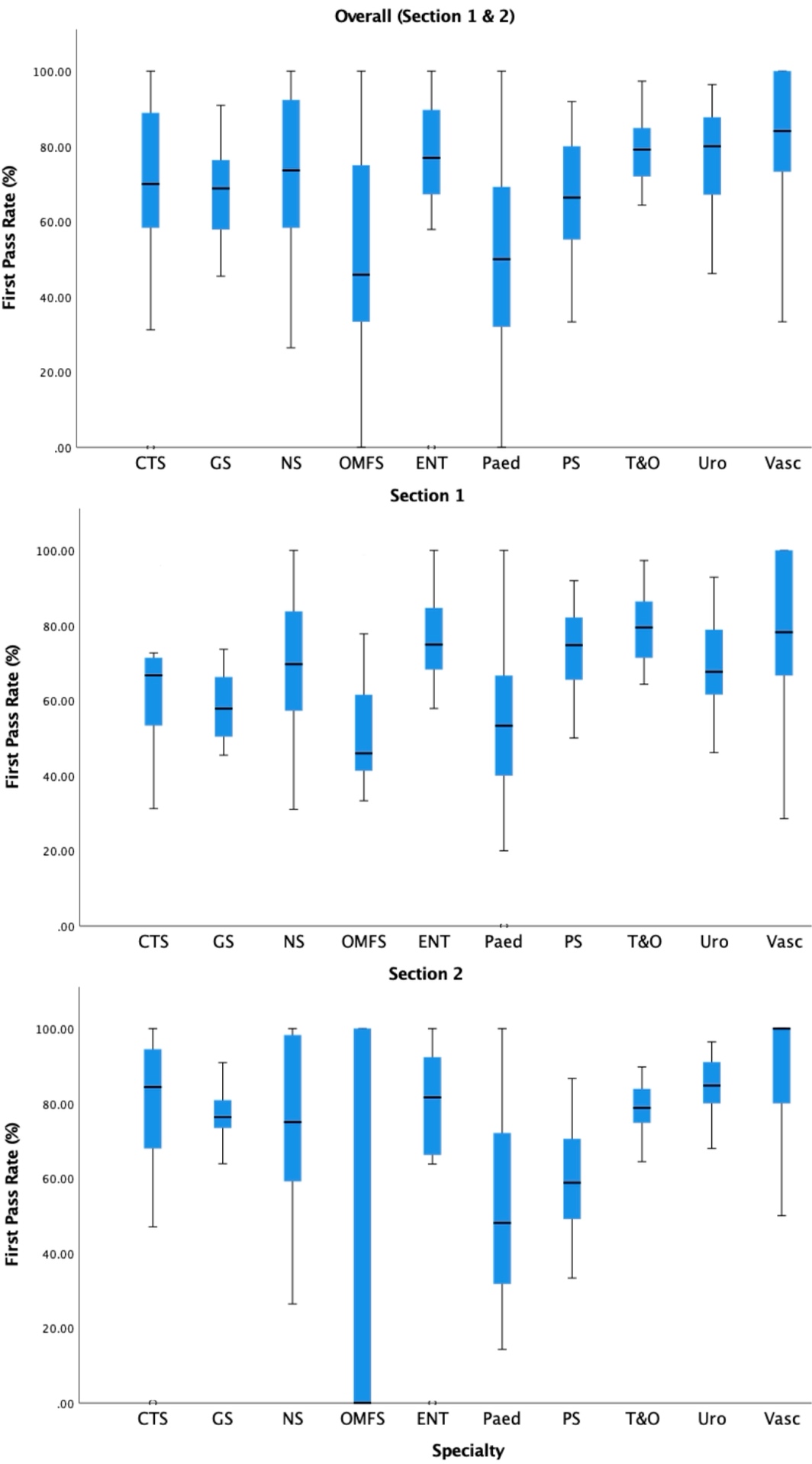
***Supplementary Figures**

**Figure S1.** FRCS first attempt pass rate by specialty.

**Supplementary Tables**

|  | **Competition Ratio** | **First Attempt Pass Rate (%)** | |
| --- | --- | --- | --- |
| **Speciality** |  | **Section 1** | **Section 2** |
| Cardiothoracic | 8.85 | 66.7 (31.3-100.0) | 84.3 (0.0-100.0) |
| Neurosurgery | 6.76 | 69.7 (31.0-100.0) | 75.0 (26.5-100.0) |
| Paediatric Surgery | 4.99 | 53.3 (0.0-100.0) | 48.1 (14.3-100.0) |
| Oral & Maxillofacial | 3.69 | 45.9 (33.3-100.0) | 0.0 (0.0-100.0) |
| Plastic Surgery | 3.68 | 74.8 (50.0-91.9) | 58.8 (33.3-86.7) |
| Trauma & Orthopaedics | 3.33 | 79.5 (64.3-97.3) | 78.8 (64.4-89.7) |
| Vascular Surgery | 3.21 | 78.2 (28.6-100.0) | 100.0 (33.3-100.0) |
| Otolaryngology | 3.09 | 74.9 (25.0-100.0) | 81.6 (0.0-100.0) |
| General Surgery | 2.67 | 57.9 (45.5-73.7) | 76.3 (63.9-90.9) |
| Urology | 2.17 | 67.6 (46.2-92.9) | 84.8 (60.0-96.4) |
| rho | | -0.273 | -0.430 |
| p-value | | 0.446 | 0.214 |

**Table S1.** Competition ratios and FRCS success by speciality.
